# Thick shells and medially wedged posts increase foot orthoses medial longitudinal arch stiffness: an experimental study

**DOI:** 10.1186/s13047-023-00609-z

**Published:** 2023-03-03

**Authors:** Ana Sofia Tavera Pelaez, Nader Farahpour, Ian B. Griffiths, Gabriel Moisan

**Affiliations:** 1 Department of Human Kinetics, Université du Québec à Trois-Rivières, Trois-Rivières, Canada; 2 Faculty of Engineering, Universidad de Antioquia, Medellín, Colombia; 3 Department of Sport Biomechanics, Faculty of Sport Sciences, Bu Ali Sina University, Hamedan, Iran; 4 Sports and Exercise Medicine, William Harvey Research Institute, Queen Mary University of London, London, UK; 5 Groupe de Recherche sur les Affections Neuromusculosquelettiques (GRAN), Université du Québec à Trois-Rivières, Trois-Rivières, Canada

**Keywords:** Foot orthoses, Medial arch stiffness, Rigidity, Mechanical properties, Force

## Abstract

**Background:**

Foot orthoses (FOs) are commonly prescribed devices to attenuate biomechanical deficits and improve physical function in patients with musculoskeletal disorders. It is postulated that FOs provide their effects through the production of reaction forces at the foot-FOs interface. An important parameter to provide these reaction forces is their medial arch stiffness. Preliminary results suggest that adding extrinsic additions to FOs (e.g., rearfoot posts) increases their medial arch stiffness. A better understanding of how FOs medial arch stiffness can be modulated by changing structural factors is necessary to better customise FOs for patients. The objectives of this study were to compare FOs stiffness and force required to lower the FOs medial arch in three thicknesses and two models (with and without medially wedged forefoot-rearfoot posts).

**Methods:**

Two models of FOs, 3D printed in Polynylon-11, were used: (1) without extrinsic additions (mFO), and (2) with forefoot-rearfoot posts and a 6^o^ medial wedge (FO6MW). For each model, three thicknesses (2.6 mm, 3.0 mm, and 3.4 mm) were manufactured. FOs were fixed to a compression plate and vertically loaded over the medial arch at a rate of 10 mm/minute. Two-way ANOVAs and Tukey post-hoc tests with Bonferroni corrections were used to compare medial arch stiffness and force required to lower the arch across conditions.

**Results:**

Regardless of the differing shell thicknesses, the overall stiffness was 3.4 times greater for FO6MW compared to mFO (*p* < 0.001). FOs with 3.4 mm and 3.0 mm thicknesses displayed 1.3- and 1.1- times greater stiffness than FOs with a thickness of 2.6 mm. FOs with a thickness of 3.4 mm also exhibited 1.1 times greater stiffness than FOs with a thickness of 3.0 mm. Overall, the force to lower the medial arch was up to 3.3 times greater for FO6MW than mFO and thicker FOs required greater force (*p* < 0.001).

**Conclusions:**

An increased medial longitudinal arch stiffness is seen in FOs following the addition of 6^o^ medially inclined forefoot-rearfoot posts, and when the shell is thicker. Overall, adding forefoot-rearfoot posts to FOs is significantly more efficient than increasing shell thickness to enhance these variables should that be the therapeutic aim.

## Background

Lower limb musculoskeletal disorders such as plantar heel pain, posterior tibial tendon dysfunction and Achilles tendinopathy are common and affect the biomechanics of locomotion [1–4]. These disorders affect muscular activity, foot and ankle kinematics and kinetics, deteriorating the dynamic stability and balance control of affected individuals [1–4]. Foot orthoses (FOs) are devices commonly prescribed to attempt to attenuate these biomechanical deficits, improve physical function and relieve pain [5, 6]. They were historically prescribed with the belief that they restore normal medial longitudinal arch and skeletal alignment during locomotion and thus provide therapeutic benefits [7] according to Root et al. paradigm [8]. Even though FOs do provide therapeutic benefits to patients [5, 9, 10], recent systematic reviews concluded that FOs do not consistently or predictably realign lower limbs during locomotion as previously suggested [11–13]. One systematic review with meta-analysis showed that FOs reduce rearfoot eversion by approximately two degrees during gait [14], which is far from being sufficient to completely realign the rearfoot in relation to the leg.

Even though there are discrepancies between the conclusions of different studies regarding the biomechanical effects of FOs during locomotion [11–13], it has been hypothesised that they could provide their clinical benefits through their kinetic effects, according to the tissue stress model [15]. FOs modify plantar pressure [16, 17], normalise centre of pressure trajectory [18] as well as modify joint moments [17, 18]. However, most studies that investigated the effects of FOs on lower limb biomechanics have used generic devices with little customisation to individuals’ morphological and biomechanical particularities [11, 12]. Such devices may not generate sufficient reaction forces to modify lower limb biomechanics and thus be inadequate for studied populations and generate conflicting results. The number of articles quantifying the biomechanical effects of FOs rises rapidly but unfortunately, there is still little understanding on how FOs design features change the mechanics of these devices. Thus, there is an important need to study how FOs structural factors and extrinsic additions influence their ability to produce reaction forces to eventually determine how these may translate into biomechanical changes to the lower limb during functional tasks.

According to the subtalar joint axis location and rotational equilibrium theory of foot function [19], FOs provide their kinetic effects through the production of reaction forces at the foot-FOs interface. FOs medial longitudinal arch stiffness is a key parameter as greater stiffness is correlated with greater pronatory control of the foot and ankle during locomotion [16, 18, 20]. Rearfoot and forefoot extrinsic posts are among the most commonly used extrinsic additions in research and in clinical contexts [11, 13, 21, 22] to increase FOs medial arch stiffness and consequently enhance their ability to change lower limb biomechanics through greater resistance to deformation [17, 23]. FOs with posts decrease ankle eversion angles/moments [23–26] and tibialis posterior muscle activity [26] during locomotion. Preliminary results showed that adding posts to FOs increases the medial longitudinal arch stiffness by up to 35% [27]. However, even though these FOs extrinsic additions have been used for a few decades, their utilisation to modify medial arch stiffness remains an emerging rather than a proven concept.

Thus, there is a lack of accurate understanding of the interactions between the mechanical properties of FOs and the underlying treatment mechanism. Recent work suggests that increasing FOs stiffness potentiates their kinematic and kinetic effects during gait, especially by further reducing rearfoot eversion angles [16] and further increasing midfoot plantar pressure [18] compared to more compliant FOs. This demonstrates that FOs stiffness is an essential parameter to modify foot biomechanics, which should be optimally set for each individual. However, individual device stiffness parameters are ignored in the vast majority of previous studies by including prefabricated FOs or custom FOs for which the only element of customisation across participants is the individual moulding of their feet [11, 12]. For prefabricated FOs, the same material thickness and geometry, either with or without extrinsic posts, are used for participants with different foot morphologies whom could require different FOs stiffnesses to obtain the same biomechanical effects. Despite the importance of FOs stiffness, no studies, to our knowledge, quantified the stiffness of FOs made with material of different thicknesses or with and without medially wedged extrinsic rearfoot and forefoot posts. It is crucial to understand how FOs stiffness varies across different shell thicknesses, and the potential effects of medially wedged forefoot and rearfoot posts on FOs stiffness. Clinicians and researchers would ideally have access to a classification of FOs stiffness in relation to their thickness and an index to link FOs specificities to the appropriate FOs stiffness for a particular patient. This information could help design more customised FOs with greater comfort and efficacy for the patients wearing them.

To address this objective, the principal aim of this study was to compare the stiffness of FOs with three different thicknesses, in two different models (with and without medially wedged forefoot and rearfoot posts). The secondary aim was to compare the peak force required to lower the medial longitudinal arch, for a same displacement across thicknesses and models. It was hypothesised that stiffness and force will increase for thicker FOs and when adding medially wedged forefoot and rearfoot posts.

## Methods

### Foot orthoses

**Fig. 1:**
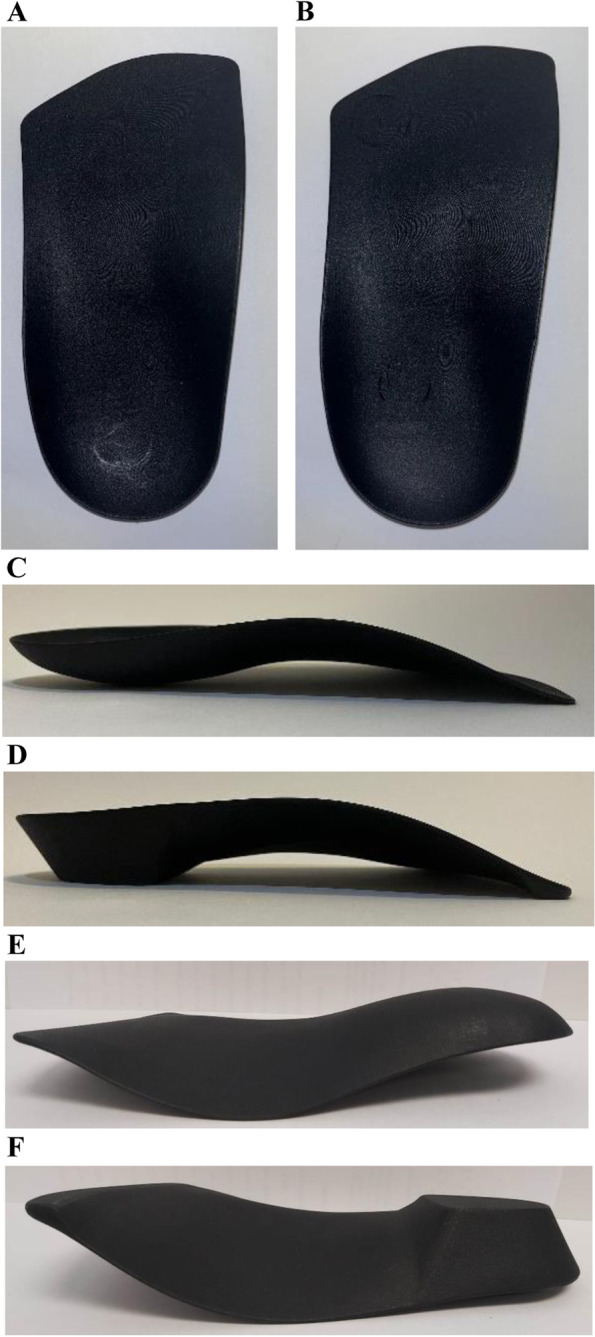
**A** FO without extrinsic additions (top), **B** FO with rearfoot and forefoot posts and a 6^o^ medial wedge (top), **C** FO without extrinsic additions (side), **D** FO with rearfoot and forefoot posts and a 6^o^ medial wedge (side), **E** FO without extrinsic additions (bottom), **F** FO with rearfoot and forefoot posts and a 6^o^ medial wedge (bottom)

A 3D scan of the right foot of a healthy male with a rectus foot type (shoe size: 44 EUR), held in subtalar joint neutral position using Root et al. [28] method, was performed using Podform3D Mobile Application (Podform3D, Montréal, Canada) based on the iPhone TrueDepth camera. The foot scan was exported as a stereolithography (STL) file. The scanned foot was processed using Podform3D CAD Software and then sent to the 3D printer. FOs were printed by Podform 3D in Polynylon-11 using an HP Multi Jet Fusion 3D printer, a powder bed fusion technology subcategory. Two models of FOs were used in this study and were chosen based on the tissue stress model [15] and subtalar joint axis location and rotational equilibrium theory of foot function (19)‘s principles: (1) without extrinsic additions (minimalist FO (mFO)), and (2) with forefoot-rearfoot posts and a 6^o^ medial wedge (FO6MW) (see Fig. 1). For each model, three shell thicknesses (2.6 mm, 3.0 mm, and 3.4 mm) were manufactured for a total of six FOs samples. All FOs had the same geometry (width, length and arch height). The only differences between experimental conditions were their thickness and the presence or absence of forefoot and rearfoot posts with a 6^o^ medial wedge:

- Condition #1: mFO, thickness of 2.6 mm.
- Condition #2: mFO, thickness of 3.0 mm.
- Condition #3: mFO, thickness of 3.4 mm.
- Condition #4: FO6MW, thickness of 2.6 mm.
- Condition #5: FO6MW, thickness of 3.0 mm.
- Condition #6: FO6MW, thickness of 3.4 mm.

### Experimental protocol

**Fig. 2:**
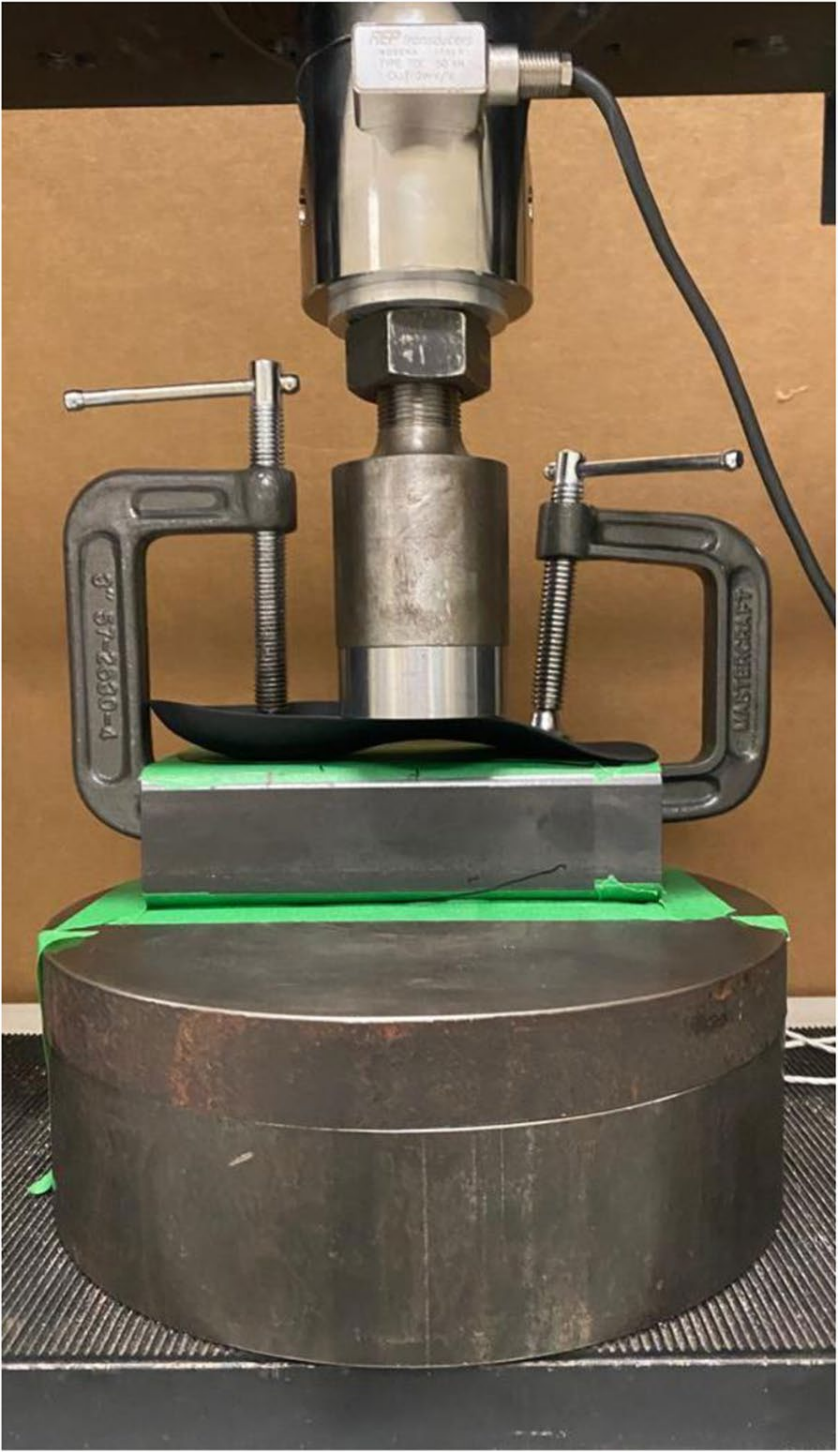
Experimental setup

To measure the stiffness of the experimental conditions, a compression test device (Instron, model 204, France) with a 60 mm diameter indenter was used. In order to standardise the compression test across conditions, each FO was firmly fixed in a horizontal position on a rectangular metal hollow compression plate using two heavy-duty G-clamps (see Fig. 2). The movable jaw of the screw of the G-clamps (circular, 2.5 cm^2^) were fixed on the FO at two ends on the points corresponding to the central point of the heel cup and 2 cm from the antero-lateral border of the FO. The FO was fixed to the compression plate in order for it not to experience any rotation while being compressed. The FO on the compression plate was properly positioned in the Instron system so that the indenter contacted the highest point of the medial edge (peak height of the arch) on its initial contact with the FO during compression (see Fig. 2). The position of the compression plate on the test device and the position of the FO on the compression plate were marked with tape to ensure the reproducibility of the measures across conditions. The dynamic compression force through the indenter was applied over the medial longitudinal arch of the FOs at a constant displacement rate of 10 mm/minute.

Starting from the zero position (initial contact with the FO), the displacement (mm) of the peak point of the medial arch and the corresponding force (N) applied by the system, were continuously recorded. The test ended when the vertical displacement of the highest point of the arch reached 5 mm. In a pilot study, using the estimated variance, a power analysis was performed to estimate a minimum required sample size. For a confidence interval of one standard deviation, with type one error of 0.05 and 0.01, four and eight trials were respectively required for each FO. However, 10 repeated trials were performed for each FO, making up to 60 trials for all FOs. Data were collected using Test Loop software (Retro-fit model number LM-U150. Compagnie Lab-Integration, Canada) and then exported into Microsoft Excel 2019 (v.16). FOs stiffness was defined as the slope of the load-displacement curves (in N/mm). Peak force required to lower the arch by 5 mm was also calculated for each FO. This value was chosen to avoid damaging the experimental conditions and ensure the reliability of the results.

### Statistical analysis

Kolmogorov-Smirnov tests were used to evaluate the normality of the distributions. As data were normally distributed, parametric tests were used. Two-way ANOVAs with two independent factors including “model” with two levels, and “thickness” with three levels; and Tukey for post-hoc tests with Bonferroni corrections were applied in SPSS-26 for stiffness and force. Bonferroni corrections were applied in statistical comparisons to limit the chances of making familywise type I statistical errors. The significance level was set at *p* = 0.050 for all comparisons.

## Results

### Stiffness

**Fig. 3:**
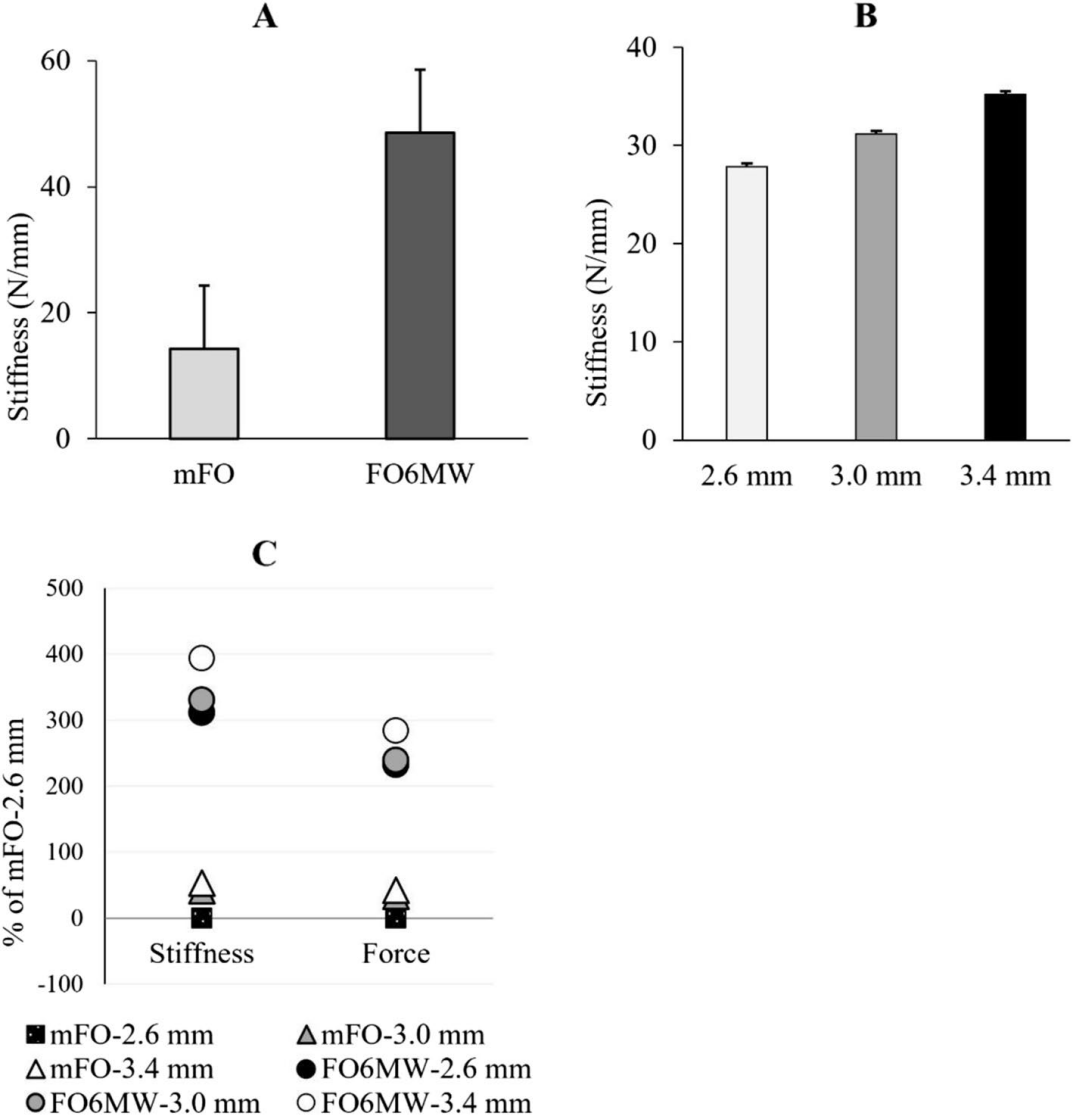
**A** Stiffness in mFO and FO6MW models regardless of the thickness effects, **B** FOs stiffness in different thicknesses, regardless of models **C** The ratio (%) of increased stiffness and force as a result of adding medially wedged forefoot-rearfoot posts, and increasing the thickness

Table 1 summarises the medial arch stiffness with different shell thicknesses for mFO and FO6MW models. Regardless of the shell thickness effects, the overall stiffness was 3.4 times greater for FO6MW compared to mFO (*F* = 37,375.4; *p* < 0.001) (see Fig. 3A). Also, the significant effects of the thickness factor revealed that the stiffness was increased with the increase of the thickness (df = 2; F 578.8; *p* < 0.001). FOs with a thickness of 3.4 mm and 3.0 mm displayed 1.3- and 1.1-times greater stiffness than FOs with a thickness of 2.6 mm. FOs with a thickness of 3.4 mm also exhibited 1.1 times greater stiffness than FOs with a thickness of 3.0 mm (see Fig. 3B).

**Table 1:** Stiffness and force required to lower the medial arch in six different FOs

Model	Thickness (mm)	Stiffness (N/mm)	Force (N)
	2.6	10.90 ± 0.35	82.15 ± 1.41
mFO	3.0	15.39 ± 0.71	109.01 ± 5.00
	3.4	16.63 ± 0.14	116.91 ± 0.57
	2.6	44.85 ± 0.96	273.43 ± 3.51
FO6MW	3.0	46.97 ± 0.43	278.30 ± 2.78
	3.4	53.85 ± 1.04	315.68 ± 7.36

Captions: Stiffness and force data are expressed as mean ± standard deviation

The significant interaction between the thickness and design features also implies that adding forefoot-rearfoot posts with a 6^o^ medial wedge multiplies the stiffness in all three thicknesses, but the effects of these additions differ based on FO thickness (see Table 1 and Fig. 3C).

### Force

**Fig. 4:**
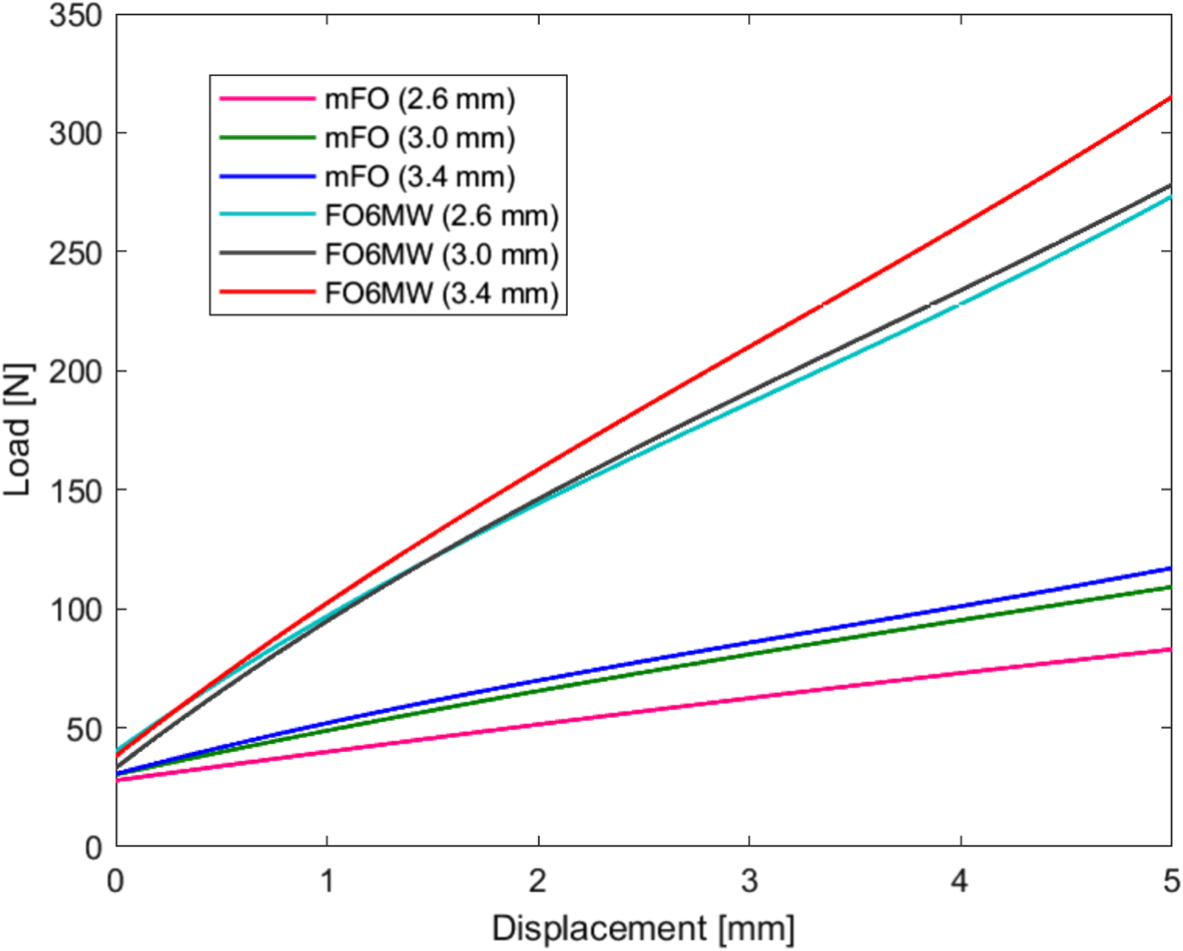
Average load-displacement curves for each FOs model and thickness

The forces required to lower the medial arch of mFO and FO6MW models in different thicknesses during the compression tests are presented in Table 1, Figs. 3C and 4. The main effect of the model factor (mFO and FO6MW) was significant (df = 1; *F* = 30,803; *p* < 0.001). Overall, FO6MW resisted compression up to 3.3 times more than mFO (*p* < 0.001). Also, the significant main effects of the thickness factor (df = 2; *F* = 442.55; *p* < 0.001) revealed that regardless of models, FOs with a thickness of 3.4 mm and 3.0 mm displayed 1.2- and 1.1-times greater force resistance than FOs with a thickness of 2.6 mm, respectively. There was a significant interaction between thickness and model factors (df = 2; *F* = 69.32; *p* = 0.001) (Fig. 3C).

Increasing mFOs thickness by 0.4 mm and 0.8 mm increased the force required to lower the medial arch by 32 and 42%, respectively (Fig. 3C). The addition of medially wedged forefoot-rearfoot posts resulted in over a 232% (over 2.3 times) greater force required to lower the medial arch compared to mFO with a thickness of 2.6 mm (Fig. 3C). Additionally, on top of the force added by the medially wedged forefoot-rearfoot posts, when the thickness of the FO6MW was increased by 0.4 mm and 0.8 mm, the force required to lower the arch was additionally increased by another 6 and 52%, respectively.

## Discussion

This study aimed to compare the medial arch stiffness and the force required to lower the medial longitudinal arch between FOs without extrinsic additions and FOs with forefoot-rearfoot posts and a 6^o^ medial wedge in three different shell thicknesses. The compression tests demonstrated that FOs with different features have highly different stiffnesses and the force required to lower the medial arch was significantly changed across conditions. The most important findings of this study were that, according to our hypothesis, 3D printed FOs in Polynylon-11 with greater thickness presented greater medial arch stiffness, and that FO6MW were significantly stiffer than mFO.

FOs thickness in the two models (mFO and FO6MW) was increased by 0.4 mm (15% relative increase) between the first and the second thickness and by 0.4 mm (13% relative increase) between the second and the third thickness. For mFO, the first increase in thickness (from 2.6 to 3.0 mm) increased its arch stiffness by 41%. The additional increase in thickness (from 3.0 to 3.4 mm) reflected in an additional 11% increase of stiffness, for a total of 52% increase between the thinner and thicker shells (2.6 vs 3.4 mm). For FO6MW, the first increase in thickness (from 2.6 mm to 3.0 mm) only increased arch stiffness by 5%, and the additional increase in thickness (from 3.0 to 3.4 mm) resulted in an additional increase of 15%, for a total increase in arch stiffness of 20% between the thinner and the thicker shells (2.6 vs 3.4 mm) (See Table 1 and Fig. 3C). These results reveal that the importance of FOs thickness to increase its stiffness is highly dependent on the presence or absence of forefoot-rearfoot posts with a 6^o^ medial wedge. As forefoot-rearfoot posts provide additional support underneath the proximal and distal part of FOs’ arch, the proportional importance of shell thickness is lower than for mFO.

Also, we found that to increase FOs medial longitudinal arch stiffness and peak force required to lower the arch, adding medially wedged forefoot-rearfoot posts was significantly more efficient than increasing shell thickness. For example, by increasing mFO’s thickness from 2.6 mm to 3.4 mm, the force required to lower the arch and the arch stiffness were only 1.4 times (117 vs 82 N) and 1.5 times (16.6 vs 10.9 N/mm) greater, respectively. However, by adding medially wedged forefoot-rearfoot posts on the 2.6 mm mFO, the force required to lower the arch and the arch stiffness were 3.3 times (273 vs 82 N) and 4.1 times (44.8 vs 10.9 N/mm) greater, respectively. These results are consistent with previous results of Griffiths et al. [27]. These authors compared the medial longitudinal arch stiffness of FOs made of a 3.0 mm thick polypropylene with and without a 4^o^ medial/varus rearfoot post. They reported a 35% increase in medial arch stiffness when adding the medial/varus post to the FOs. In our study, we observed a 205% increase in arch stiffness when adding medially inclined posts to the FOs with a thickness of 3.0 mm, rather than 35%. This greater increase could be explained by the addition of a forefoot post in our study, the greater posts inclination (6^o^ vs 4^o^), the material difference (Polynylon-11 vs polypropylene) and/or the FOs arch height and length. These results suggest that if one wants to significantly increase FOs medial arch stiffness, medially wedged forefoot-rearfoot posts should be used rather than only changing shell thickness.

### Research and clinical perspectives

Recent systematic reviews reported conflicting results pertaining to the biomechanical effects of FOs during locomotion [11–13]. For example, during gait, previous studies reported that FOs decreased rearfoot eversion [23, 25], had no effect on rearfoot eversion [6, 29] and even increased rearfoot eversion [24]. These conflicting results could be explained by the large heterogeneity of participants’ morphological specificities (e.g., foot types), musculoskeletal status and evaluated tasks across studies [11]. Based on these elements, we hypothesise that some participants may require stiffer FOs than others to exhibit significant biomechanical changes. In other words, FOs stiffness may have been insufficient for some participants and thus could explain the variability of the biomechanical responses across participants and studies. For example, individuals with a posterior tibial tendon dysfunction exhibit greater rearfoot eversion angles and internal inversion moments compared to healthy counterparts [4, 30]. Chicoine et al. [25] found that stiffer FOs including forefoot-rearfoot posts and a 5^o^ medial wedge were sufficient to attenuate rearfoot eversion angles and internal inversion moments in participants with a posterior tibial tendon dysfunction whereas more compliant FOs without posts had no effects on these variables. Overall, there is an important need to study the relationships between FOs stiffness and biomechanical effects during locomotion, comfort and eventually their influence on pain, function and clinical outcomes.

### Limitations

The results of this study should be interpreted within the context of some limitations. Firstly, the compression test we performed primarily focused on the stiffness at the FOs’ medial longitudinal arch peak height. This test represents an oversimplification of the in-vivo loading during which the FOs medial arch is loaded from different directions, in all regions of the arch, rather than only on the peak edge. The compression tests did not account for shoes worn by patients with their FOs and which could modify loading parameters. Also, in the present experimental protocol, both the rearfoot and lateral forefoot of the orthosis were clamped to the mechanical test machine which may have overestimated the medial arch stiffness. Without the lateral forefoot clamp, the point of the application of the force on the orthosis would be changed due to the rotational reaction of the forefoot of the orthosis under the pressure resulting in poor reliability of the data. The distal clamp prevented the frontal rotation of the distal part of FOs with the cost of a possible overestimation of the arch stiffness. However, this effect is minimized with the fact that the same condition was applied for all testing condition. Future studies would ideally evaluate medial arch stiffness in different FOs models and thicknesses using an indenter matching in-vivo loading, for example with a simulated foot.

Secondly, the load applied to the FOs medial arch may not be representative of the force applied during locomotion. For example, Aminian et al. [31] reported a mean maximum force under the medial midfoot (i.e., the medial arch) of 2.46 N/kg. In their study, participants’ mean body mass was 72.9 kg and thus the mean absolute force on the FOs medial arch during gait would be approximately 179 N. In our study, we observed values ranging from 82 to 117 N for mFO and 273 to 316 N for FO6MW. The applied forces to FO6MW may perhaps not be representative of in-vivo loading. However, during locomotion the peak force under the midfoot is averaged across a larger surface [31]. The lower values of force under the centre of the foot could lower the average for the entire medial midfoot and thus explain the higher values found in our study. In a study using a similar compression test to evaluate the load-deformation curves of FOs, the authors reported values of force of approximately 250–300 N to lower the arch of 5 mm [32], which is consistent with our results. However, care must be taken before extrapolating our results to FOs of other models, thicknesses, arch heights, materials and also before translating our results into clinical recommendations to practitioners.

## Conclusions

Adding forefoot and rearfoot posts with a 6^o^ medial wedge and increasing shell thickness will increase FOs medial longitudinal arch stiffness and the peak force required to lower the arch. Overall, adding medially wedged forefoot-rearfoot posts to FOs has a greater influence on these variables than increasing shell thickness. Future studies should attempt to quantify the stiffness of the medial arch and force required to lower the arch in FOs with other design features and correlate these variables with FOs biomechanical effects during locomotion.

## Data Availability

The datasets used and/or analysed during the current study are available from the corresponding author on reasonable request.

## Abbreviations

FO6MW: Foot orthosis with a 6^o^ medial wedge
FOs: Foot orthoses
mFO: Minimalist foot orthosis
